# Cocaine

**Published:** 1897-06

**Authors:** W. I. Jones

**Affiliations:** Nelsonville, Ohio.


ARTICLE IV.

### COCAINE.

BY DR. W. I. JONES, NELSONVILLE, OHIO.

The following is a formula we use and pronounce good:

| | | |
|---|---|---|
| ℞ | Cocaine, | gr. x. |
| | Listerine, | ℥ ss. |
| | Aquæ distillat., | ℥ ij. |

This makes a little less than one and a half per cent. solution. In fifteen minims of this soultion, which will be found sufficient for the extraction of a tooth, there is but a fraction over a fifth of a grain; this amount is, with rare exceptions, non-toxic. We have used cocaine for over seven years, and in the last two years we have injected it hypodermically into more than two thousand persons, have used it in numerous combinations, various percentages, and on "all sorts and conditions of men." We have had some peculiar experiences, all of them interesting and instructive. Very rarely will a careful use of cocaine cause any untoward result; ordinarily it is quickly eliminated. Sometimes it will stimulate, and a sense of well-being is produced; at other times the injection is followed by a feeling of oppression, which may be quickly relieved by the inhalation of ammonia or ether. After an over-dose the pulse becomes weak and rapid, breathing is labored and shallow, a cold perspiration breaks out, and the skin is clammy. These symptoms are at times accompanied by clonic couvulsions. In a case of this kind nothing should be done that will excite the patient. He should be placed in a recumbent position, and stimulants administered. Aromatic spirit of ammonia is one of the most reliable cardiac stimulants, given by the mouth in fifteen to twenty minim doses, diluted in water or injected hypodermically; inhaled, it stimulates the trifacial nerve and increases blood-tension by reflex action on the vaso-motor center, and prevents syncope. To relieve the nervous symptoms, one-eighth grain doses of morphine may be given by the mouth. If the patient is unable to swallow, inject hypodermically.

Great care should at all times be used in the administration of an anesthetic, no less so with a local anesthetic than with one of general effect. We have given cocaine when the patient was known to be affected with valvular disease of the heart, believing the heroic method for the extraction of a tooth in a case of this kind more liable to do harm than would cocaine, and we have administered large doses to persons on whom at first minute doses have had apparently toxic effects; the eyes would become dull, countenance pale, skin clammy, which was quickly relieved by stimulants, more cocain given, and as the operation proceeded the patient grew stronger. Idiosyncrasy, no doubt is the only explanation for many peculiar cases. By the injection of pure water I have caused what was apparently cocaine poisoning. If cocaine had been used, it would have been condemned for what was only a manifestation of the temperature of the patient.

Eighteen months ago William R. came to my office to have a tooth extracted. Cacaine was injected, and almost immediately we found it necessary to administer stimulants. A year later he returned to have another extracted, and requested that "that stuff" should not be used, thinking that it had made him sick the last time. We complied, and turned to get an instrument. This opened the show; and there followed every indication of cocaine poisoning.

If a hysterical woman should come to your office accompanied by a hen-pecked husband, and you should hnve a scene, do not be in a hurry to attribute it to cocaine. In every case study the temperament of your patient, and act accordingly.—*Dental Cosmos.*

